# Monitoring Changes in Salivary Amino Acids in Smokers Using Nanofiber-Based Solid-Phase Extraction Combined With High-Performance Liquid Chromatography

**DOI:** 10.7759/cureus.102104

**Published:** 2026-01-22

**Authors:** Kangwei Shen, Wei Tang, Changjian Wu, Hai Zhao, Ahad Hussain, Yan Yan, Yi Cao, Wei Fan, Mengjie Li, Qing Lu, Xuejun Kang, Penglin Wu

**Affiliations:** 1 School of Biological Science and Medical Engineering, Southeast University, Nanjing, CHN; 2 Pharmacy, Zhongda Hospital, Southeast University, Nanjing, CHN; 3 Analytical Chemistry, China Tobacco Jiangsu Industrial Co. Ltd, Nanjing, CHN; 4 School of Public Health, Southeast University, Nanjing, CHN

**Keywords:** 4-ethylenedioxythiophene nanofiber, dansyl chloride, high-performance liquid chromatography, poly-3, salivary amino acid, smoking, solid phase extraction

## Abstract

Introduction: Smoking causes a variety of diseases and is the primary cause of preventable death. Smoking can cause metabolic disorders of amino acids (AAs) in body fluids. However, the trace concentration of AAs and complex background interference in biological samples pose a challenge for the determination of AAs. Conventional solid-phase extraction for the concentration and purification of AAs is often ineffective because AAs are highly polar.

Materials and methods: We selected poly-3,4-ethylenedioxythiophene (PEDOT) nanofibers as adsorbents for the extraction of AAs from saliva after derivatization with dansyl chloride. Thirteen AAs in saliva were determined by high-performance liquid chromatography (HPLC) with an ultraviolet detector. The proposed method was used to determine the dynamic changes of AA concentrations in the saliva of six subjects before and after smoking.

Results: Under the optimized conditions, adjusting the acidity of the sample solution to pH 2 and the loading amount of PEDOT nanofibers to 18 mg, the established method provided a quantitative limit of 0.15 to 0.45 micrograms per milliliter for the 13 AAs, with a recovery rate of 61.8% to 130.2% and a precision of 3.6-12.0%. Compared with the no-smoking blank, the areas under the concentration-time curves for glycine and alanine in saliva were significantly increased (p< 0.05). The concentrations of these two AAs in saliva reached their peak 15 minutes after smoking, suggesting that these two AAs may potentially play a role in the mechanism of the smoking effect.

Conclusions: The method, developed using PEDOT nanofibers for solid-phase extraction of AAs coupled with HPLC, thereby improving detection sensitivity and reducing interference, was suitable for the quantitative determination of AAs in saliva. It was found that smoking caused significant dynamic changes in salivary glycine and alanine.

## Introduction

As people's awareness of health continues to rise, the relationship between smoking and health has drawn increasing public attention. Research shows that smoking is one of the risk factors for non-communicable diseases such as cardiovascular diseases, cancer, chronic lung diseases, and diabetes. There are over 5,000 chemical components in tobacco products and cigarette smoke, among which more than 150 have been identified as harmful substances, making it challenging to select exposure biomarkers [1]. The smoke from cigarettes is a complex mixture of various components, and the pathological conditions caused by this mixture are also very complex. The mechanisms of action and causes of these diseases are currently poorly understood. Smoking can cause an imbalance of amino acids (AAs) in the smoker's body, thereby damaging the function of the respiratory system. These metabolic disorders largely contribute to the occurrence and development of smoking-related diseases (such as cancer and cardiovascular diseases) [2]. Analysis of AAs in biological samples may help identify specific biomarkers and molecular pathways associated with smoking-related metabolic disorders.

A variety of techniques have been employed for the determination of AAs, including high-performance liquid chromatography (HPLC), gas chromatography/mass spectrometry, capillary electrophoresis, etc. [3]. Due to a lack of strong chromophores or fluorophores in their molecules, the determination of AAs usually requires the derivatization of these compounds before measurement. The derivatization agents used include 6-aminoquinolinyl-N-hydroxysuccinimidyl carbamate, o-phthalaldehyde, phenyl isothiocyanate, 9-fluorenylmethyl chloroformate, 2,4-dinitrofluorobenzene, etc. [4]. However, the extremely low levels of AAs and the complex interfering substances in biological samples pose significant challenges for detection [5].

AAs are a class of polar substances, and the recovery rate is poor when using solid-phase extraction for concentration and purification. This might be the main reason why concentration and purification steps are rarely performed in AA analysis [6]. The reported solid-phase extraction methods usually employ ion-exchange columns. Due to the need for several milliliters of eluent to release the target compounds, multiple steps are often required, such as evaporation, drying, and re-dissolution, resulting in a relatively long processing time. Some researchers have also developed novel nanomaterials for extracting AAs from complex samples, such as zeolite imidazolate framework-8 (ZIF-8) nanoparticles and functionalized graphene oxide [5]. However, these two studies mainly focused on the extraction of aromatic AAs.

Our research group has successfully extracted polar molecules from biological samples using conductive polymer nanofiber materials, such as short-chain fatty acids [7], cysteine derivatives [8], and tryptophan metabolites [9]. We have also established an efficient liquid chromatography detection method for the simultaneous detection of 13 AAs derived from dansyl chloride (dimethylamino naphthalene-sulfonyl chloride (Dns)) [10]. Since the concentration characteristics of AAs in saliva can reflect the physiological and psychological states of primates [11], and saliva samples are non-invasive and easy to collect, this study aims to use conductive polymer nanofibers for the solid-phase extraction of 13 AAs from saliva. After derivatization with Dns, 13 AAs, including arginine (Arg), ornithine (Orn), lysine (Lys), glutamine (Gln), serine (Ser), hydroxyproline (Hyp), glutamic acid (Glu), aspartic acid (Asp), glycine (Gly), threonine (Thr), taurine (Tau), alanine (Ala), and gamma aminobutyric acid (GABA), were detected using liquid chromatography with an ultraviolet detector, and the changes in saliva AA profiles after volunteers smoked cigarettes were monitored. Try to see if it's possible to initially identify biochemical markers of smoking behavior in saliva.

## Materials and methods

Reagents and chemicals

Methanol (chromatographic purity) was purchased from Chengdu Xindu Industrial Development Zone (Chengdu, China). Triethyl ammonium was from Sinopharm Chemical Reagent Co., Ltd. Tetrabutyl ammonium hydroxide was from Shanghai Kefeng Industry Co., Ltd. (Shanghai, China). Dansyl chloride and 13 AA standards were from Sigma (St. Louis, MO). Acetone was from Shanghai Lingfeng Chemical Reagent Co., Ltd. (Shanghai, China). Potassium bicarbonate, concentrated sulfuric acid, ethanol, potassium hydroxide, and glacial acetic acid were from Nanjing Chemical Reagent Co., Ltd. (Jiangsu Province, China). Polystyrene (PS) nanofiber, polypyrrole (PPY) nanofiber, poly-3,4-ethylenedioxythiophene (PEDOT) particles, and nanofibers were from Suzhou Donqi Biotechnology Co., Ltd. (Jiangsu Province, China). Triply distilled water was used for all studies. Artificial saliva was from Yuanye Bio-Technology Co., Ltd. (Shanghai, China).

Accurately weighed 10 mg of each AA (Arg, Orn, Lys, Gln, Ser, Hyp, Glu, Asp, Gly, Thr, Tau, Ala, and GABA) and placed them in 10 ml volumetric flasks, respectively, and dissolved each of them with triply distilled water to prepare 1.0 mg/mL stock standard solution separately. These solutions were stored in a brown flask at 4°C for later use and diluted daily to working concentrations with triply distilled water. The dansyl chloride solution was prepared just before derivatization by dissolving 200 mg of Dns in 10 mL of acetone.

Instruments

SHIMADZU LC-20AD HPLC (Shimadzu Corporation); SPD-10AD UV detector (Shimadzu Corporation); LC-20AD Double pump; SIL-20AC Autosampler; CTO-20A Column Compartment; METTLERAT electronic analytical balance; BF-2500 positive and negative pressure oil-free vacuum pump (Shanghai Rate Nako Trading Co., Ltd.); TGL-16 high-speed centrifuge (Jintan Ronghua Instrument Manufacturing Co., Ltd.); XW-80A vortex mixer (Shanghai Qingpu Huxi Instrument factory); Hitachi S-3000N scanning electron microscope (SEM; Japan's Hitachi); PHS-2C pH meter (Shanghai Weiye); Shimadzu C18 column (4.6×150 mm, 5μm); Electric thermostatic water bath (Jintan Ronghua Instrument Manufacturing Co., Ltd.). Barometric solid-phase extraction instrument (Suzhou Donqi Biotechnology Co., Ltd.).

Preparation of the extraction column

**Figure 1:**
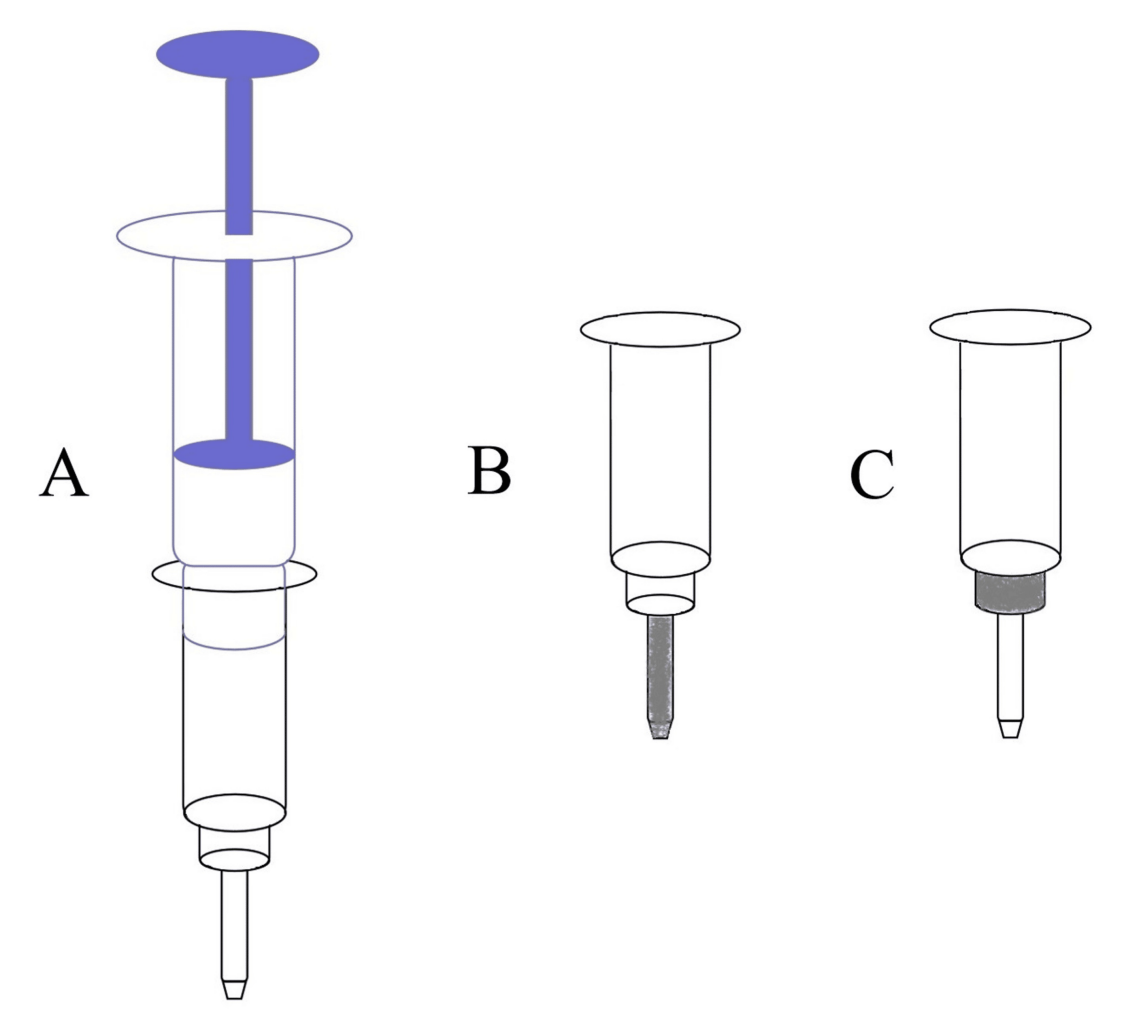
Schematic representation for the solid-phase extraction column (A) Solid-phase extraction device, (B) cylindrical extraction column, and (C) disc-shaped solid-phase extraction column Image Credit: Authors

The solid-phase extraction device shown in Figure 1A was used in this study. For the PEDOT particle adsorbent, a small amount of glass wool should be pre-filled at the tube tip to prevent particles from leaking out. For the fibrous adsorbent, no glass wool needs to be packed; weigh an appropriate amount of nanofibers and use a thin steel rod (with a diameter of approximately 0.5 millimeters) to fill the nanofibers into the thinnest part (diameter 2.5 mm) of the cartridge to form a cylindrical extraction column (Figure 1B). To investigate the effect of column shape on extraction efficiency, PEDOT nanofibers were packed into the slightly thicker section of the cartridge (6 mm in diameter), forming a disc-shaped solid-phase extraction column (Figure 1C). Before extraction, the columns should be washed with 100 μL methanol and 200 μL distilled water, slowly.

Participants' recruitment and sample acquisition

Six volunteers who have a smoking habit were recruited to participate in the experiment. On the first day, they smoked a commercially available cigarette (taking two Marlboro cigarettes in succession). On the second day, the samples without smoking were set as the blank control. The experiment started at 9:00 in the morning. The samples were collected before smoking and 5, 15, 30, 45, and 60 minutes after smoking. Approximately 1 mL of naturally secreted saliva was collected and stored at -20 °C for processing. This study has been approved by the Institutional Ethics Committee of Zhongda Hospital, Southeast University (approval number: 2024ZDSYLL326-P01). Written informed consent was obtained from all participants.

Sample handling

The frozen saliva sample was thawed and vortexed for one minute, then centrifuged at 12,000 rpm for five minutes. 200 μL of the supernatant was mixed with 10 μL of 1.2 mol/L hydrochloric acid, then transferred to the solid-phase extraction column. The liquid was slowly pressed out drop by drop using a barometric solid-phase extraction instrument, allowing the target compounds to be adsorbed onto the sorbent. The absorbed target substances were then eluted from the column with 100 μL of methanol. The eluent was then added to 50 μL of a buffer solution containing 2 mol/L KOH-KHCO₃ (pH 9.8) and 50 μL of Dns solution (20 mg/mL). After capping the tube and vigorously mixing for five seconds, the mixture was heated to 60℃ in a water bath for 60 minutes in the dark. Then, an aliquot (20 μL) of acetic acid was added to the tube to stop the reaction. The mixture was centrifuged at 10000 g for five minutes. The 20 μL supernatant of the reaction mixture was injected into the HPLC system.

Chromatographic conditions

The mobile phase was 35:65 methanol/water (V/V) containing 1.5% triethylammonia and 5 mmol/L tetrabutylammonium hydroxide at pH 2.5 (adjusted with concentrated sulfuric acid). The UV detection wavelength was 221 nm. The flow rate of the mobile phase was 1.5 mL/min. The injection volume was 20 µL. The column temperature was 35℃.

## Results

Adsorbent morphological characterization and performance screening

**Figure 2:**
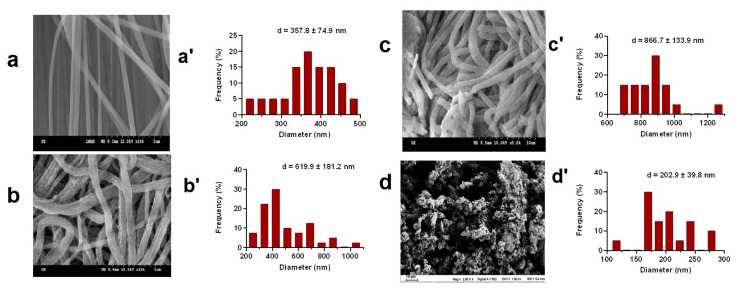
SEM images of nanomaterials and diameter distributions (a, a’) PS nanofiber, (b, b’) PEDOT nanofiber, (c, c’) PPY nanofiber, (d, d’) PEDOT particle SEM: scanning electron microscope, PS: polystyrene, PEDOT: poly-3,4-ethylenedioxythiophene, PPY: polypyrrole

**Figure 3:**
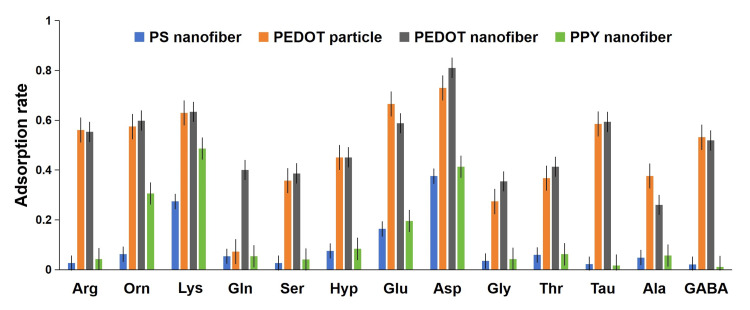
Comparison of the adsorption effect of AAs on different materials Blue: PS nanofibers, orange: PEDOT particles, black: PEDOT nanofibers, green: PPY nanofibers AAs: amino acids, PS: polystyrene, PEDOT: poly-3,4-ethylenedioxythiophene, PPY: polypyrrole, Arg: arginine, Orn: ornithine, Lys: lysine, Gln: glutamine, Ser: serine, Hyp: hydroxyproline, Glu: glutamic acid, Asp: aspartic acid, Gly: glycine, Thr: threonine, Tau: taurine, Ala: alanine, GABA: gamma-aminobutyric acid

SEM images and diameter distributions (Figure 2) show that PEDOT and PPY nanofibers, fabricated by polymerizing monomers on PS nanofiber templates, have larger average diameters than PS nanofibers. All three fibrous materials maintain a highly porous network structure, while PEDOT nanoparticles exhibit a non-fibrous morphology that requires filter plates or additional fillers for column packing. Carry out three repetitions of the adsorption efficiency tests (Figure 3) to demonstrate that PS nanofibers generally have lower adsorption capacities for 13 AAs compared to the other three materials. PEDOT-based adsorbents (nanofibers and particles) outperform PPY nanofibers, and PEDOT nanofibers achieve comparable adsorption efficiency to PEDOT particles without the need for extra packing materials, leading to their selection for subsequent experiments.

Optimization of key extraction conditions (column shape, pH, and adsorbent loading)

**Figure 4:**
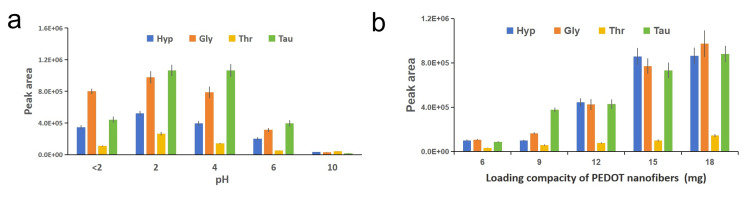
Influence of the different parameters on the extraction (a) pH of saliva, (b) loading capacity of PEDOT nanofibers PEDOT: poly-3,4-ethylenedioxythiophene, Hyp: hydroxyproline, Gly: glycine, Thr: threonine, Tau: taurine

Three repeated optimization experiments on extraction conditions reveal significant effects of column shape, pH, and adsorbent loading. Cylindrical columns (diameter 2.5 mm) exhibit higher extraction efficiencies for four representative AAs (Hyp, Gly, Thr, Tau) than disc-shaped columns (diameter 6 mm), with the disc-shaped column efficiencies ranging from 0.191 to 0.677 when the cylindrical column efficiency is set to 1 (Table 1). Maximum extraction efficiencies for all tested AAs are achieved at pH 2 (Figure 4A), with gradual decreases observed at pH values above or below this optimal point. In terms of adsorbent loading, extraction efficiencies increase with PEDOT nanofiber dosage from 6 mg to 18 mg (Figure 4B), and 18 mg is selected as the optimal amount to balance extraction efficiency, cartridge volume constraints, and the risk of analyte dilution by eluent.

**Table 1:** Comparison of the extraction efficiency of AAs using different columns AAs: amino acids, Hyp: hydroxyproline, Gly: glycine, Thr: threonine, Tau: taurine

Compounds	Cylindrical column	Disc-shaped column
Hyp	1	0.32
Gly	1	0.248
Thr	1	0.191
Tau	1	0.677

Systematic validation of the analytical method

**Figure 5:**
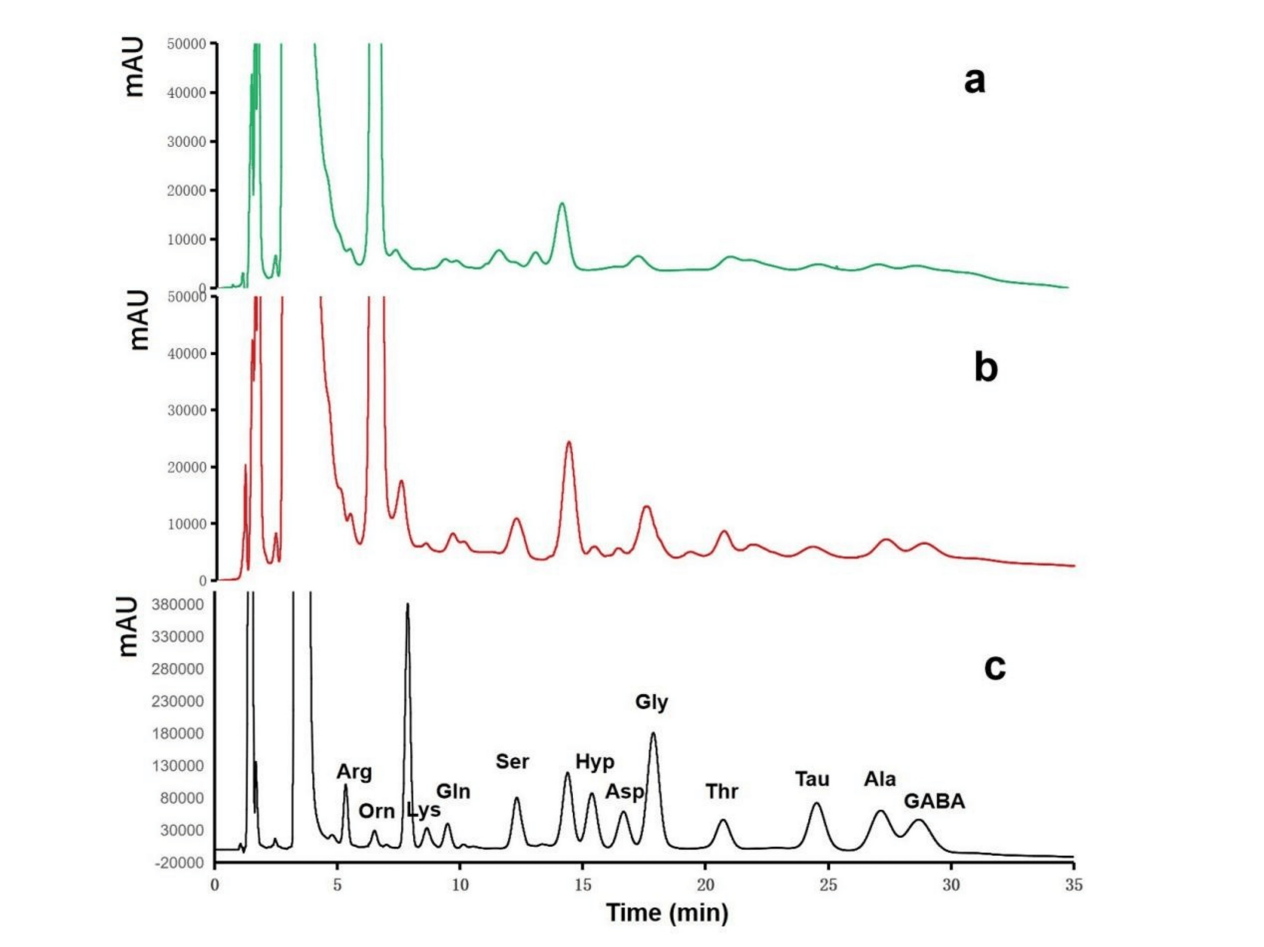
Chromatograms of saliva samples (a) Saliva sample without extraction, (b) saliva sample with extraction by PEDOT nanofibers, (c) standard solution (100μg/mL) PEDOT: poly-3,4-ethylenedioxythiophene, Arg: arginine, Orn: ornithine, Lys: lysine, Gln: glutamine, Ser: serine, Hyp: hydroxyproline, Glu: glutamic acid, Asp: aspartic acid, Gly: glycine, Thr: threonine, Tau: taurine, Ala: alanine, GABA: gamma-aminobutyric acid

Method validation results confirm the reliability of the optimized approach. Thirteen AAs show good linearity in the range of 0.5-100 μg/mL (1.0-100 μg/mL for some analytes) with correlation coefficients (R²) greater than 0.991 (Table 2). Limits of detection (LOD, S/N = 3) range from 0.05 to 0.15 μg/mL, and limits of quantification (LOQ, S/N = 10) are between 0.15 and 0.45 μg/mL. Recoveries at three spiked concentration levels (1, 5, 10 μg/mL) vary from 61.8% to 130.2% with relative standard deviations (RSD%) of 3.6-12.0% (Table 2). Chromatograms (Figure 5) indicate that target AA peaks are significantly enhanced after extraction with PEDOT nanofibers, and the elution curves are consistent with those of standard solutions, demonstrating effective elimination of matrix interference.

**Table 2:** Linear ranges, correlation coefficients, LOD, LOQ, and recoveries of 18 AAs R²: coefficient of determination, LOD: limit of detection, LOQ: limit of quantification, RSD: relative standard deviation, AAs: amino acids, Arg: arginine, Orn: ornithine, Lys: lysine, Gln: glutamine, Ser: serine, Hyp: hydroxyproline, Glu: glutamic acid, Asp: aspartic acid, Gly: glycine, Thr: threonine, Tau: taurine, Ala: alanine, GABA: gamma-aminobutyric acid

AAs	Linear range (μg/mL)	R^2^ value	Spiked concentration (μg/mL, n = 3)						LOD (μg/mL)	LOQ (ug/mL)
			10		5		1			
			Recovery(%)	RSD (%)	Recovery(%)	RSD (%)	Recovery(%)	RSD (%)		
											Arg	0.5-100	0.999	104.6	5.9	72.9	6.2	66.7	8.9	0.05	0.15
Orn	0.5-100	0.996	120.6	5.7	123.4	7.6	112.6	10.1	0.05	0.15
Lys	1.0-100	0.993	115.3	7.3	69.2	12.0	115.1	11.0	0.15	0.45
Gln	0.5-100	0.997	89.0	9.2	121.4	7.0	89.6	11.9	0.05	0.2
Ser	0.5-100	0.999	95.3	5.8	130.2	6.0	83.7	6.4	0.05	0.15
Hyp	0.5-100	0.999	61.8	4.9	82.5	6.6	99.9	7.8	0.05	0.15
Glu	0.5-100	0.998	85.8	3.6	106.1	5.8	70.7	8.2	0.05	0.2
Asp	0.5-100	0.992	70.1	4.9	102.7	6.6	127.3	11.8	0.05	0.15
Gly	0.5-100	0.998	82.7	7.1	93.3	10.7	124.0	10.7	0.05	0.15
Thr	1.0-100	0.991	120.6	5.4	123.4	7.9	111.7	11.8	0.15	0.4
Tau	0.5-100	0.998	101.2	3.6	112.5	8.2	112.9	10.1	0.05	0.3
Ala	0.5-100	0.997	102.0	4.5	116.8	6.7	114.9	7.8	0.05	0.2
GABA	0.5-100	0.993	95.6	4.0	108.5	5.4	70.7	8.2	0.05	0.2

Variation of salivary AAs in smoking

**Figure 6:**
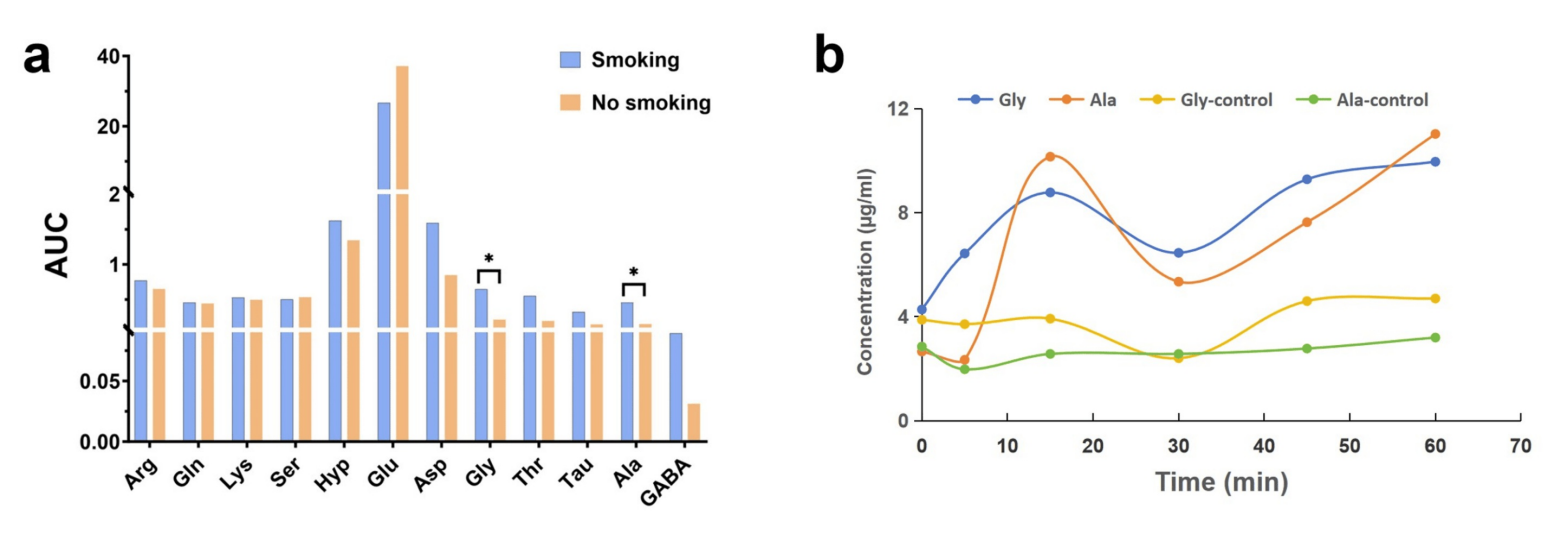
Changes in salivary AAs before and after smoking (a) Comparison of the area under saliva concentration-time curves, (b) saliva concentration-time curves of glycine and alanine * p < 0.05 indicates statistical significance. One-way ANOVA was used to analyze differences between the two groups of AAs. AAs: amino acids, AUC: area under saliva concentration-time curves, ANOVA: analysis of variance, PEDOT: poly-3,4-ethylenedioxythiophene, Arg: arginine, Orn: ornithine, Lys: lysine, Gln: glutamine, Ser: serine, Hyp: hydroxyproline, Glu: glutamic acid, Asp: aspartic acid, Gly: glycine, Thr: threonine, Tau: taurine, Ala: alanine, GABA: gamma-aminobutyric acid

To verify the feasibility of this method for analyzing saliva samples, we used it to determine changes in AA concentrations in saliva from healthy volunteers before and after smoking. The concentrations of various AAs at each of the five time points before and after smoking were plotted against time, and the area under the curve (AUC) of the graphs was calculated using GraphPad Prism 10.1.2 (GraphPad Software, LLC, San Diego, CA, USA) as a parameter for differential analysis. The results are shown in Figure 6a. Compared with the blank control, only the AUCs of Gly and Ala in the saliva concentration-time curves showed significant differences (p < 0.05), whereas the AUCs of other AAs differed but did not reach statistical significance. After smoking, the concentrations of Gly and Ala in saliva increased for up to 15 minutes, reaching a peak, as shown in Figure 6b.

Smoking can activate the hypothalamic-pituitary-adrenal axis and the autonomic nervous system. The most commonly used research methods assess changes in cortisol and plasma catecholamine levels (i.e., adrenaline and noradrenaline) in saliva or blood, as well as cardiovascular responses, including systolic and diastolic blood pressure, heart rate, and heart rate variability [12]. However, as far as we know, few studies report whether the levels of saliva AAs were also changed after smoking.

Gly is a non-essential AA that can be synthesized from Ser. Gly is an inhibitory neurotransmitter that plays a crucial role in inhibitory synapses in the spinal cord, brainstem, and cerebellum, as well as in excitatory synapses throughout the brain. It is related to the neurobehavioral phenomenology of alcohol use disorders [13]. Ala is also a non-essential AA that can be synthesized by the human body or obtained from food. It plays a significant role in protein synthesis, energy metabolism, and neurotransmitter production. In the body, Ala can be converted into other AAs through transamination, such as glutamate. Glutamate is an important excitatory neurotransmitter that plays a crucial role in the central nervous system. It participates in synaptic transmission and affects learning, memory, and cognitive functions. Through conversion into glutamate, Ala indirectly participates in the synthesis and release of neurotransmitters [14]. Ala has been reported to regulate the anxiety-like behavior of rats [15]. This study found that the levels of Gly and Ala in saliva significantly changed after smoking, providing a new perspective for exploring the mechanism of the physiological and psychological effects of smoking on the human body.

## Discussion

Correlation between adsorbent structure and adsorption performance

The structure of adsorbents directly influences their adsorption performance. PS nanofibers, relying on hydrophobic interactions, fail to effectively adsorb polar AAs. At the same time, conductive polymers (PEDOT and PPY) polymerized on PS templates not only retain the highly porous fibrous structure (providing abundant active sites) but also enhance adsorption through hydrogen bonding (between oxygen-containing groups of the polymers and AA functional groups) and electrostatic interactions. PEDOT-based materials exhibit superior performance compared to PPY nanofibers, and PEDOT nanofibers offer greater practicality than PEDOT particles by eliminating the need for additional packing materials (glass wool). The template polymerization process successfully achieves both "structure retention and function enhancement" for the adsorbents.

Influencing mechanisms of optimized extraction conditions

The optimization of extraction conditions aims primarily to maximize the effective interactions between adsorbents and target analytes. Cylindrical columns provide a longer bed depth, extending the contact time between samples and adsorbents and increasing the probability of interaction, whereas the shorter flow path of disc-shaped columns results in insufficient contact and lower extraction efficiency. At pH 2, AAs are protonated, matching the surface charge characteristics of PEDOT nanofibers and thereby maximizing hydrogen-bonding strength. Deviations from pH 2 alter the charge states of either AAs or the adsorbent, disrupting these interactions. Increasing adsorbent loading directly increases the number of active sites, but excessive loading requires more eluent, leading to analyte dilution. The selection of 18 mg PEDOT nanofibers balances extraction efficiency, cartridge volume limitations, and elution effectiveness.

Practicality and application value of the established method

The established method demonstrates excellent practicality and application value. Low LOD and LOQ values enable the detection of trace AAs in saliva, while good linearity and reproducibility (RSD% <12%) ensure quantitative accuracy. The wide recovery range (61.8-130.2%) adapts to the complexity of the saliva matrix. The highly porous structure and specific interactions of PEDOT nanofibers not only improve extraction efficiency but also effectively purify the matrix, resulting in significantly enhanced chromatographic peaks for target AAs. Overall, through adsorbent screening, extraction condition optimization, and systematic validation, this method provides a reliable approach for the efficient enrichment and accurate quantification of 13 AAs in saliva, offering a technical reference for related biological sample analysis.

Comparison with other methods

The number of studies on the pretreatment of AAs using solid-phase extraction is limited. Table 3 presents a comparison of parameters between this study and several other studies. Most earlier studies used ion-exchange resins as adsorbents, which require elution with polar media (such as water); concentrating the eluent by nitrogen blowing is quite time-consuming. As shown in Table 3, literature 5, 4, and 17 did not specify the time required for the nitrogen blowing, so the time consumed in the extraction process was not included in the table. Compared with other methods, the solid-phase extraction technique established in this study was time-efficient and required less organic solvent, indicating that the proposed method was more environmentally friendly and labor-saving. The other parameters, such as recovery, LOD, and precision, also met the analytical requirements. Using PEDOT nanofibers solved the problem of difficulty in concentrating and enriching target substances for the detection of AAs in complex samples.

**Table 3:** Comparative information from some studies on pretreatment and detection of AAs in samples AAs: amino acids, LOD: limit of detection, RSD: relative standard deviation, LC-MS: liquid chromatography-mass spectrometry, IC: ion chromatography, HPLC: high-performance liquid chromatography, CE: capillary electrophoresis, IE: ion-exchange, ZIF-8: zeolite imidazolate framework-8, —a: not specified in the literature

Number of AAs	Matrices	Method	Adsorbent	Organic solvent (mL)	Sample volume (mL)	Extraction	Relative recovery (%)	LOD	Precision RSD%	Evaporate and redissolve	References
						time					
						(min)					
15	Water	LC-MS	IE resin	12.5	—	>30	69.8-117.9	0.01-0.27 nmol/L	0.3-13.2	Yes	6
14	Serum	IC	IE resin	0	0.2	>15	80.6-114.8	0.02～0.59μｇ/mＬ	1.9-10.4	Yes	16
26	Urine	LC-MS	IE resin	10	0.1	— ^a^	88.2-117.0	LOD ≤1.4 µmol /L	<10	Yes	17
3	Blood	CE	ZIF-8	9	1	26	90.1-95.0	0.13-0.37 μg /mL	<8.1	No	3
6	Lycium barbarum	HPLC	C18	10.8	1	— ^a^	87.3-97.1	2.42～6.51 μmol/L	2.62-5.22	Yes	4
3	Urine	HPLC	GO-MONs-NH2	3.5	2	— ^a^	90.8-107.5	3.96-7.13 ng/mL	≤6.4	Yes	5
13	Saliva	HPLC	PEDOT	0.2	0.2	5	69.2-124.0	0.05-0.15μg /mL	3.6-12.0	No	This work
			Nanofiber								

Limitations

There were several obvious limitations in this study, including the recruitment of only six volunteers for the experiment, all male and aged 30-40. There was no strict control over other potential confounding factors, such as diet and oral health conditions, which led to statistical bias. There were only 13 AAs to be determined; some important AA variables may have been missed. The number of subjects should be expanded further to determine the characteristics of changes in human salivary AAs.

## Conclusions

Using HPLC-ultraviolet detection combined with solid-phase extraction, a method for determining and quantifying 13 AAs in saliva was successfully developed and verified. The optimized PEDOT electrospun nanofibers were used for solid-phase extraction of AAs in saliva, significantly enhancing the signal intensity of the target peaks. The extraction process did not require steps such as solvent evaporation and re-dissolution, as in classical ion-exchange solid-phase extraction, reducing labor intensity, contamination, and the amount of organic solvents. The extracted AAs were derivatized with Dns before HPLC analysis. This method was applied to the identification and quantification of salivary AAs and to the exploration of their dynamic changes in saliva after smoking. It was found that glycine and alanine in saliva were gradually increased to peak values at 15 minutes after smoking, and the areas under the saliva concentration-time curves of these two AAs were significantly higher compared to the non-smoking samples. This discovery provided new ideas for studying the physiological and psychological effects of smoking, and the established method has potential for AA analysis in biological samples.
